# Supplementary material for: Comparative multiomics analysis of cell physiological state after culture in a basket bioreactor
Source: Sci Rep. 2022 Nov 23;12:20161. doi: 10.1038/s41598-022-24687-4 (PMC9686226; doi:10.1038/s41598-022-24687-4)
Supplement: Supplementary file 1 — Supplementary Information 1. [file 41598_2022_24687_MOESM1_ESM.zip › raw data/Proteomic raw data/6.KEGGenrichment/EG.vs.CG/UP/pathway/KEGG.map.html]

Pathway Enrichment


  

# The most enriched pathway terms of EG.vs.CG

Statistic method: hypergeometric test

FDR correction method: Benjamini and Hochberg

| MapID | MapTitle | Pvalue | AdjustedPv | x | y | n | N | EnrichDirect | ProtID | Description  | |
| --- | --- | --- | --- | --- | --- | --- | --- | --- | --- | --- | --- |
| map04110 | Cell cycle | 0.00010634927115561 | 0.0121238169117395 | 6 | 58 | 39 | 2848 | Over | A0A0D9REV4 A0A0D9RJF7 A0A0D9RKU0 A0A0D9RLR8 A0A0D9RQN8 A0A0D9RYS3 | Uncharacterized protein,Structural maintenance of chromosomes protein 1A,DNA replication licensing factor MCM3,Proliferating cell nuclear antigen,DNA replication licensing factor MCM4,Uncharacterized protein |
| map03030 | DNA replication | 0.000361698645554795 | 0.0206168227966233 | 4 | 26 | 39 | 2848 | Over | A0A0D9RKU0 A0A0D9RLR8 A0A0D9RQN8 A0A0D9RWI0 | DNA replication licensing factor MCM3,Proliferating cell nuclear antigen,DNA replication licensing factor MCM4,Replication factor C subunit 1 |
| map00072 | Synthesis and degradation of ketone bodies | 0.00485784149669244 | 0.184597976874313 | 2 | 8 | 39 | 2848 | Over | A0A0D9RJH6 A0A0D9S9P1 | Uncharacterized protein,Uncharacterized protein |
| map01522 | Endocrine resistance | 0.0176766113718207 | 0.282384659141731 | 3 | 41 | 39 | 2848 | Over | A0A0D9R491 A0A0D9RRV8 A0A0D9RYS3 | Uncharacterized protein,Bile salt export pump,Uncharacterized protein |
| map05225 | Hepatocellular carcinoma | 0.0181725743790765 | 0.282384659141731 | 4 | 75 | 39 | 2848 | Over | A0A0D9R491 A0A0D9R7G0 A0A0D9RYS3 A0A0D9SCP4 | Uncharacterized protein,Glutathione transferase,Uncharacterized protein,Uncharacterized protein |
| map05220 | Chronic myeloid leukemia | 0.0213516488536326 | 0.282384659141731 | 3 | 44 | 39 | 2848 | Over | A0A0D9R1V2 A0A0D9R491 A0A0D9RYS3 | E3 ubiquitin-protein ligase CBL,Uncharacterized protein,Uncharacterized protein |
| map00900 | Terpenoid backbone biosynthesis | 0.0218317563533567 | 0.282384659141731 | 2 | 17 | 39 | 2848 | Over | A0A0D9RJH6 A0A0D9S9P1 | Uncharacterized protein,Uncharacterized protein |
| map00650 | Butanoate metabolism | 0.0243505760523442 | 0.282384659141731 | 2 | 18 | 39 | 2848 | Over | A0A0D9RJH6 A0A0D9S9P1 | Uncharacterized protein,Uncharacterized protein |
| map03430 | Mismatch repair | 0.0269826929001285 | 0.282384659141731 | 2 | 19 | 39 | 2848 | Over | A0A0D9RLR8 A0A0D9RWI0 | Proliferating cell nuclear antigen,Replication factor C subunit 1 |
| map04950 | Maturity onset diabetes of the young | 0.0272048637832846 | 0.282384659141731 | 1 | 2 | 39 | 2848 | Over | A0A0D9QYX8 | Uncharacterized protein |
| map00380 | Tryptophan metabolism | 0.0297247009622875 | 0.282384659141731 | 2 | 20 | 39 | 2848 | Over | A0A0D9RJH6 A0A0D9S9P1 | Uncharacterized protein,Uncharacterized protein |
| map04979 | Cholesterol metabolism | 0.0297247009622875 | 0.282384659141731 | 2 | 20 | 39 | 2848 | Over | A0A0D9RJC1 A0A0D9RRV8 | Epididymal secretory protein E1,Bile salt export pump |
| map00630 | Glyoxylate and dicarboxylate metabolism | 0.0449685878169873 | 0.394339923933581 | 2 | 25 | 39 | 2848 | Over | A0A0D9RJH6 A0A0D9S9P1 | Uncharacterized protein,Uncharacterized protein |
| map00600 | Sphingolipid metabolism | 0.0517235771407521 | 0.396816113716893 | 2 | 27 | 39 | 2848 | Over | A0A0D9QUW7 A0A0D9RHA9 | Glucuronosyltransferase,Aminotran\_1\_2 domain-containing protein |
| map03013 | RNA transport | 0.0597442329292335 | 0.396816113716893 | 4 | 109 | 39 | 2848 | Over | A0A0D9QZ96 A0A0D9QZT6 A0A0D9R2B7 A0A0D9RYG7 | RanBD1 domain-containing protein,RRM domain-containing protein,Uncharacterized protein,Death domain-containing protein |
| map05161 | Hepatitis B | 0.0600814703132367 | 0.396816113716893 | 3 | 66 | 39 | 2848 | Over | A0A0D9R491 A0A0D9RLR8 A0A0D9RYS3 | Uncharacterized protein,Proliferating cell nuclear antigen,Uncharacterized protein |
| map05218 | Melanoma | 0.0624865643201884 | 0.396816113716893 | 2 | 30 | 39 | 2848 | Over | A0A0D9R491 A0A0D9RYS3 | Uncharacterized protein,Uncharacterized protein |
| map00071 | Fatty acid degradation | 0.0662311939929364 | 0.396816113716893 | 2 | 31 | 39 | 2848 | Over | A0A0D9RJH6 A0A0D9S9P1 | Uncharacterized protein,Uncharacterized protein |
| map00640 | Propanoate metabolism | 0.0662311939929364 | 0.396816113716893 | 2 | 31 | 39 | 2848 | Over | A0A0D9RJH6 A0A0D9S9P1 | Uncharacterized protein,Uncharacterized protein |
| map03420 | Nucleotide excision repair | 0.0700498211991536 | 0.396816113716893 | 2 | 32 | 39 | 2848 | Over | A0A0D9RLR8 A0A0D9RWI0 | Proliferating cell nuclear antigen,Replication factor C subunit 1 |
| map00620 | Pyruvate metabolism | 0.0778986895222709 | 0.396816113716893 | 2 | 34 | 39 | 2848 | Over | A0A0D9RJH6 A0A0D9S9P1 | Uncharacterized protein,Uncharacterized protein |
| map00310 | Lysine degradation | 0.0860129490896834 | 0.396816113716893 | 2 | 36 | 39 | 2848 | Over | A0A0D9RJH6 A0A0D9S9P1 | Uncharacterized protein,Uncharacterized protein |
| map01212 | Fatty acid metabolism | 0.0860129490896834 | 0.396816113716893 | 2 | 36 | 39 | 2848 | Over | A0A0D9RJH6 A0A0D9S9P1 | Uncharacterized protein,Uncharacterized protein |
| map05223 | Non-small cell lung cancer | 0.0860129490896834 | 0.396816113716893 | 2 | 36 | 39 | 2848 | Over | A0A0D9R491 A0A0D9RYS3 | Uncharacterized protein,Uncharacterized protein |
| map04910 | Insulin signaling pathway | 0.0893891132737259 | 0.396816113716893 | 3 | 78 | 39 | 2848 | Over | A0A0D9R1V2 A0A0D9R2B7 A0A0D9R491 | E3 ubiquitin-protein ligase CBL,Uncharacterized protein,Uncharacterized protein |
| map04150 | mTOR signaling pathway | 0.0947744219975698 | 0.396816113716893 | 3 | 80 | 39 | 2848 | Over | A0A0D9QZT6 A0A0D9R2B7 A0A0D9SCP4 | RRM domain-containing protein,Uncharacterized protein,Uncharacterized protein |
| map04550 | Signaling pathways regulating pluripotency of stem cells | 0.102960926658696 | 0.396816113716893 | 2 | 40 | 39 | 2848 | Over | A0A0D9QYX8 A0A0D9SCP4 | Uncharacterized protein,Uncharacterized protein |
| map05215 | Prostate cancer | 0.102960926658696 | 0.396816113716893 | 2 | 40 | 39 | 2848 | Over | A0A0D9R491 A0A0D9RYS3 | Uncharacterized protein,Uncharacterized protein |
| map05200 | Pathways in cancer | 0.106918804319991 | 0.396816113716893 | 5 | 186 | 39 | 2848 | Over | A0A0D9R1V2 A0A0D9R491 A0A0D9R7G0 A0A0D9RYS3 A0A0D9SCP4 | E3 ubiquitin-protein ligase CBL,Uncharacterized protein,Glutathione transferase,Uncharacterized protein,Uncharacterized protein |
| map01524 | Platinum drug resistance | 0.111758284275433 | 0.396816113716893 | 2 | 42 | 39 | 2848 | Over | A0A0D9R491 A0A0D9R7G0 | Uncharacterized protein,Glutathione transferase |
| map04012 | ErbB signaling pathway | 0.111758284275433 | 0.396816113716893 | 2 | 42 | 39 | 2848 | Over | A0A0D9R1V2 A0A0D9R491 | E3 ubiquitin-protein ligase CBL,Uncharacterized protein |
| map00280 | Valine, leucine and isoleucine degradation | 0.116230229755852 | 0.396816113716893 | 2 | 43 | 39 | 2848 | Over | A0A0D9RJH6 A0A0D9S9P1 | Uncharacterized protein,Uncharacterized protein |
| map03040 | Spliceosome | 0.120601608863287 | 0.396816113716893 | 3 | 89 | 39 | 2848 | Over | A0A0D9QYJ1 A0A0D9RJ55 A0A0D9RYG7 | Serine/arginine-rich splicing factor 2,Uncharacterized protein,Death domain-containing protein |
| map05212 | Pancreatic cancer | 0.120748257417863 | 0.396816113716893 | 2 | 44 | 39 | 2848 | Over | A0A0D9R491 A0A0D9RYS3 | Uncharacterized protein,Uncharacterized protein |
| map01521 | EGFR tyrosine kinase inhibitor resistance | 0.125310351700072 | 0.396816113716893 | 2 | 45 | 39 | 2848 | Over | A0A0D9R2B7 A0A0D9R491 | Uncharacterized protein,Uncharacterized protein |
| map05224 | Breast cancer | 0.125310351700072 | 0.396816113716893 | 2 | 45 | 39 | 2848 | Over | A0A0D9RYS3 A0A0D9SCP4 | Uncharacterized protein,Uncharacterized protein |
| map00220 | Arginine biosynthesis | 0.128992177281689 | 0.39743535703007 | 1 | 10 | 39 | 2848 | Over | A0A0D9QZS1 | Alanine transaminase |
| map01200 | Carbon metabolism | 0.139140115969865 | 0.417420347909596 | 3 | 95 | 39 | 2848 | Over | A0A0D9QZS1 A0A0D9RJH6 A0A0D9S9P1 | Alanine transaminase,Uncharacterized protein,Uncharacterized protein |
| map04310 | Wnt signaling pathway | 0.14871431215303 | 0.434703373985779 | 2 | 50 | 39 | 2848 | Over | A0A0D9R633 A0A0D9SCP4 | Uncharacterized protein,Uncharacterized protein |
| map05217 | Basal cell carcinoma | 0.15277075750293 | 0.435396658883349 | 1 | 12 | 39 | 2848 | Over | A0A0D9SCP4 | Uncharacterized protein |
| map05205 | Proteoglycans in cancer | 0.158604540902278 | 0.440997991777065 | 3 | 101 | 39 | 2848 | Over | A0A0D9QZT6 A0A0D9R1V2 A0A0D9SCP4 | RRM domain-containing protein,E3 ubiquitin-protein ligase CBL,Uncharacterized protein |
| map05226 | Gastric cancer | 0.163165792405684 | 0.442878579386856 | 2 | 53 | 39 | 2848 | Over | A0A0D9RYS3 A0A0D9SCP4 | Uncharacterized protein,Uncharacterized protein |
| map05166 | HTLV-I infection | 0.172039244330596 | 0.455783353029167 | 3 | 105 | 39 | 2848 | Over | A0A0D9RLR8 A0A0D9RYS3 A0A0D9SCP4 | Proliferating cell nuclear antigen,Uncharacterized protein,Uncharacterized protein |
| map01210 | 2-Oxocarboxylic acid metabolism | 0.175916381870907 | 0.455783353029167 | 1 | 14 | 39 | 2848 | Over | A0A0D9QZS1 | Alanine transaminase |
| map00982 | Drug metabolism - cytochrome P450 | 0.187256982120389 | 0.464071651341835 | 1 | 15 | 39 | 2848 | Over | A0A0D9R7G0 | Glutathione transferase |
| map03410 | Base excision repair | 0.187256982120389 | 0.464071651341835 | 1 | 15 | 39 | 2848 | Over | A0A0D9RLR8 | Proliferating cell nuclear antigen |
| map00670 | One carbon pool by folate | 0.198445467011779 | 0.481335813603037 | 1 | 16 | 39 | 2848 | Over | A0A0D9RSM3 | Methenyltetrahydrofolate cyclohydrolase |
| map05219 | Bladder cancer | 0.20948382392793 | 0.497524081828833 | 1 | 17 | 39 | 2848 | Over | A0A0D9RYS3 | Uncharacterized protein |
| map02010 | ABC transporters | 0.220374014979435 | 0.502452754153111 | 1 | 18 | 39 | 2848 | Over | A0A0D9RRV8 | Bile salt export pump |
| map04610 | Complement and coagulation cascades | 0.220374014979435 | 0.502452754153111 | 1 | 18 | 39 | 2848 | Over | A0A0D9RFF4 | Coagulation factor V |
| map00250 | Alanine, aspartate and glutamate metabolism | 0.241717623441116 | 0.519920925892211 | 1 | 20 | 39 | 2848 | Over | A0A0D9QZS1 | Alanine transaminase |
| map00565 | Ether lipid metabolism | 0.241717623441116 | 0.519920925892211 | 1 | 20 | 39 | 2848 | Over | A0A0D9QUW7 | Glucuronosyltransferase |
| map05014 | Amyotrophic lateral sclerosis (ALS) | 0.241717623441116 | 0.519920925892211 | 1 | 20 | 39 | 2848 | Over | A0A0D9R491 | Uncharacterized protein |
| map04151 | PI3K-Akt signaling pathway | 0.250907691457059 | 0.52268967148392 | 3 | 127 | 39 | 2848 | Over | A0A0D9QZT6 A0A0D9R2B7 A0A0D9R491 | RRM domain-containing protein,Uncharacterized protein,Uncharacterized protein |
| map00980 | Metabolism of xenobiotics by cytochrome P450 | 0.2521748415054 | 0.52268967148392 | 1 | 21 | 39 | 2848 | Over | A0A0D9R7G0 | Glutathione transferase |
| map05204 | Chemical carcinogenesis | 0.262491495619758 | 0.534357687511649 | 1 | 22 | 39 | 2848 | Over | A0A0D9R7G0 | Glutathione transferase |
| map04014 | Ras signaling pathway | 0.274294773847636 | 0.548589547695271 | 2 | 75 | 39 | 2848 | Over | A0A0D9R491 A0A0D9RQM0 | Uncharacterized protein,Uncharacterized protein |
| map04530 | Tight junction | 0.279434067305044 | 0.54923247711681 | 2 | 76 | 39 | 2848 | Over | A0A0D9R9F6 A0A0D9RLR8 | Uncharacterized protein,Proliferating cell nuclear antigen |
| map04727 | GABAergic synapse | 0.30238892979437 | 0.572744927984833 | 1 | 26 | 39 | 2848 | Over | A0A0D9R329 | Aa\_trans domain-containing protein |
| map04120 | Ubiquitin mediated proteolysis | 0.315331883994465 | 0.572744927984833 | 2 | 83 | 39 | 2848 | Over | A0A0D9R1V2 A0A0D9RFM8 | E3 ubiquitin-protein ligase CBL,RING-type domain-containing protein |
| map00270 | Cysteine and methionine metabolism | 0.321541012202011 | 0.572744927984833 | 1 | 28 | 39 | 2848 | Over | A0A0D9R4V8 | DNA (cytosine-5)-methyltransferase |
| map04976 | Bile secretion | 0.321541012202011 | 0.572744927984833 | 1 | 28 | 39 | 2848 | Over | A0A0D9RRV8 | Bile salt export pump |
| map05221 | Acute myeloid leukemia | 0.321541012202011 | 0.572744927984833 | 1 | 28 | 39 | 2848 | Over | A0A0D9R491 | Uncharacterized protein |
| map04721 | Synaptic vesicle cycle | 0.340180417420284 | 0.58758435736231 | 1 | 30 | 39 | 2848 | Over | A0A0D9S2L0 | Uncharacterized protein |
| map05213 | Endometrial cancer | 0.340180417420284 | 0.58758435736231 | 1 | 30 | 39 | 2848 | Over | A0A0D9R491 | Uncharacterized protein |
| map04370 | VEGF signaling pathway | 0.349312058201196 | 0.589600636084741 | 1 | 31 | 39 | 2848 | Over | A0A0D9R491 | Uncharacterized protein |
| map00480 | Glutathione metabolism | 0.358320517459327 | 0.589600636084741 | 1 | 32 | 39 | 2848 | Over | A0A0D9R7G0 | Glutathione transferase |
| map04013 | MAPK signaling pathway - fly | 0.3672074137019 | 0.589600636084741 | 1 | 33 | 39 | 2848 | Over | A0A0D9RQM0 | Uncharacterized protein |
| map04916 | Melanogenesis | 0.3672074137019 | 0.589600636084741 | 1 | 33 | 39 | 2848 | Over | A0A0D9SCP4 | Uncharacterized protein |
| map05214 | Glioma | 0.3672074137019 | 0.589600636084741 | 1 | 33 | 39 | 2848 | Over | A0A0D9RYS3 | Uncharacterized protein |
| map04660 | T cell receptor signaling pathway | 0.384622887934246 | 0.608986239229223 | 1 | 35 | 39 | 2848 | Over | A0A0D9R1V2 | E3 ubiquitin-protein ligase CBL |
| map05203 | Viral carcinogenesis | 0.390826493457309 | 0.610331784303194 | 2 | 98 | 39 | 2848 | Over | A0A0D9R491 A0A0D9RYS3 | Uncharacterized protein,Uncharacterized protein |
| map05120 | Epithelial cell signaling in Helicobacter pylori infection | 0.401571019598397 | 0.614810494304565 | 1 | 37 | 39 | 2848 | Over | A0A0D9R9F6 | Uncharacterized protein |
| map04212 | Longevity regulating pathway - worm | 0.40987366286971 | 0.614810494304565 | 1 | 38 | 39 | 2848 | Over | A0A0D9QXD1 | Fatty acyl-CoA reductase |
| map04724 | Glutamatergic synapse | 0.40987366286971 | 0.614810494304565 | 1 | 38 | 39 | 2848 | Over | A0A0D9R329 | Aa\_trans domain-containing protein |
| map05202 | Transcriptional misregulation in cancer | 0.426143595114224 | 0.622040511871246 | 1 | 40 | 39 | 2848 | Over | A0A0D9RJ75 | Runt-related transcription factor |
| map05211 | Renal cell carcinoma | 0.426143595114224 | 0.622040511871246 | 1 | 40 | 39 | 2848 | Over | A0A0D9R491 | Uncharacterized protein |
| map05210 | Colorectal cancer | 0.434113822959859 | 0.622040511871246 | 1 | 41 | 39 | 2848 | Over | A0A0D9R491 | Uncharacterized protein |
| map05165 | Human papillomavirus infection | 0.441851901329897 | 0.622040511871246 | 3 | 142 | 39 | 2848 | Over | A0A0D9R491 A0A0D9RYS3 A0A0D9SCP4 | Uncharacterized protein,Uncharacterized protein,Uncharacterized protein |
| map04211 | Longevity regulating pathway | 0.441976153171675 | 0.622040511871246 | 1 | 42 | 39 | 2848 | Over | A0A0D9R2B7 | Uncharacterized protein |
| map04514 | Cell adhesion molecules (CAMs) | 0.457382793399779 | 0.635873639604571 | 1 | 44 | 39 | 2848 | Over | A0A0D9R9F6 | Uncharacterized protein |
| map05222 | Small cell lung cancer | 0.464929894347499 | 0.638578409103794 | 1 | 45 | 39 | 2848 | Over | A0A0D9RYS3 | Uncharacterized protein |
| map04670 | Leukocyte transendothelial migration | 0.479718501348107 | 0.643387166513931 | 1 | 47 | 39 | 2848 | Over | A0A0D9R9F6 | Uncharacterized protein |
| map05100 | Bacterial invasion of epithelial cells | 0.479718501348107 | 0.643387166513931 | 1 | 47 | 39 | 2848 | Over | A0A0D9R1V2 | E3 ubiquitin-protein ligase CBL |
| map05145 | Toxoplasmosis | 0.501157431325342 | 0.6581200322668 | 1 | 50 | 39 | 2848 | Over | A0A0D9R491 | Uncharacterized protein |
| map04722 | Neurotrophin signaling pathway | 0.514969227936628 | 0.6581200322668 | 1 | 52 | 39 | 2848 | Over | A0A0D9R491 | Uncharacterized protein |
| map04390 | Hippo signaling pathway | 0.521734678619915 | 0.6581200322668 | 1 | 53 | 39 | 2848 | Over | A0A0D9SCP4 | Uncharacterized protein |
| map05160 | Hepatitis C | 0.541484000771622 | 0.6581200322668 | 1 | 56 | 39 | 2848 | Over | A0A0D9R491 | Uncharacterized protein |
| map04024 | cAMP signaling pathway | 0.547888772967148 | 0.6581200322668 | 1 | 57 | 39 | 2848 | Over | A0A0D9R491 | Uncharacterized protein |
| map04114 | Oocyte meiosis | 0.547888772967148 | 0.6581200322668 | 1 | 57 | 39 | 2848 | Over | A0A0D9RJF7 | Structural maintenance of chromosomes protein 1A |
| map05206 | MicroRNAs in cancer | 0.547888772967148 | 0.6581200322668 | 1 | 57 | 39 | 2848 | Over | A0A0D9R4V8 | DNA (cytosine-5)-methyltransferase |
| map01230 | Biosynthesis of amino acids | 0.554206342961516 | 0.6581200322668 | 1 | 58 | 39 | 2848 | Over | A0A0D9QZS1 | Alanine transaminase |
| map04022 | cGMP-PKG signaling pathway | 0.554206342961516 | 0.6581200322668 | 1 | 58 | 39 | 2848 | Over | A0A0D9R491 | Uncharacterized protein |
| map04066 | HIF-1 signaling pathway | 0.554206342961516 | 0.6581200322668 | 1 | 58 | 39 | 2848 | Over | A0A0D9R2B7 | Uncharacterized protein |
| map05418 | Fluid shear stress and atherosclerosis | 0.554206342961516 | 0.6581200322668 | 1 | 58 | 39 | 2848 | Over | A0A0D9R7G0 | Glutathione transferase |
| map04919 | Thyroid hormone signaling pathway | 0.57862750704041 | 0.679790340592425 | 1 | 62 | 39 | 2848 | Over | A0A0D9R491 | Uncharacterized protein |
| map04146 | Peroxisome | 0.590344243146053 | 0.679790340592425 | 1 | 64 | 39 | 2848 | Over | A0A0D9QXD1 | Fatty acyl-CoA reductase |
| map05152 | Tuberculosis | 0.590344243146053 | 0.679790340592425 | 1 | 64 | 39 | 2848 | Over | A0A0D9R491 | Uncharacterized protein |
| map04071 | Sphingolipid signaling pathway | 0.612833097083317 | 0.698629730674981 | 1 | 68 | 39 | 2848 | Over | A0A0D9RHA9 | Aminotran\_1\_2 domain-containing protein |
| map04360 | Axon guidance | 0.623621783410098 | 0.703889933750011 | 1 | 70 | 39 | 2848 | Over | A0A0D9RQM0 | Uncharacterized protein |
| map04141 | Protein processing in endoplasmic reticulum | 0.679116282331223 | 0.759012315546661 | 2 | 120 | 39 | 2848 | Over | A0A0D9R887 A0A0D9RFU7 | DnaJ heat shock protein family (Hsp40) member B12,Derlin |
| map04140 | Autophagy - animal | 1 | 1 | 1 | 73 | 39 | 2848 | Over | A0A0D9R491 | Uncharacterized protein |
